# Axillary schwannoma mimicking lymph node metastasis-associated breast cancer: a case report

**DOI:** 10.1186/s40792-022-01493-8

**Published:** 2022-07-18

**Authors:** Hideki Kumagai, Kou Takehana, Yoshihiro Shioi, Chihiro Tono

**Affiliations:** Department of General Surgery, Iwate Prefectural Senmaya Hospital, 32-1 Kusaizawa, Ichinoseki, Iwate 029-0803 Japan

**Keywords:** Axillary schwannoma, Breast cancer, Lymph node metastasis

## Abstract

**Background:**

Axillary schwannoma associated with breast cancer is an extremely rare disease, and previous reports have been limited. In this setting, there is great concern about whether a tumor in the axillary region is lymph node metastasis. Herein, we report a unique case of axillary schwannoma that mimicked lymph node metastasis associated with breast cancer.

**Case presentation:**

A 68-year-old woman who underwent mastectomy and axillary lymph node dissection for right breast cancer over 20 years ago presented to our hospital with numbness and weakness in the right arm for 6 months. Ultrasonography, computed tomography, and magnetic resonance imaging showed a 20-mm well-circumscribed round tumor in the right axillary region. Initially, she was suspected of having lymph node metastasis-associated breast cancer, but the result of the core needle biopsy was a schwannoma. The patient underwent tumor enucleation. The patient has had no recurrence 1 year after the operation.

**Conclusion:**

Axillary schwannomas often mimic lymph node metastasis in patients with a history of malignancy, particularly breast cancer. To select the optimal treatment, the clinicians should make as accurately as possible a diagnosis, with histopathological examinations, when examining patients with cancer who develop tumors in the axillary region.

## Background

Schwannomas are the most common type of peripheral nerve tumors, but their incidence is low and comprises one per 50,000 individuals [1]. In addition, only 5% of sporadic peripheral nerve schwannomas occur in the upper extremities [2]. To the best of our knowledge, patients with an axillary schwannoma associated with breast cancer are extremely rare, and the previous reports are also limited [3–8]. In the clinical setting, there is great concern about whether such a tumor in the axillary region is a lymph node metastasis.

Herein, we report an extremely rare case of axillary schwannoma that mimicked lymph node metastasis associated with breast cancer.

## Case presentation

**Fig. 1:**
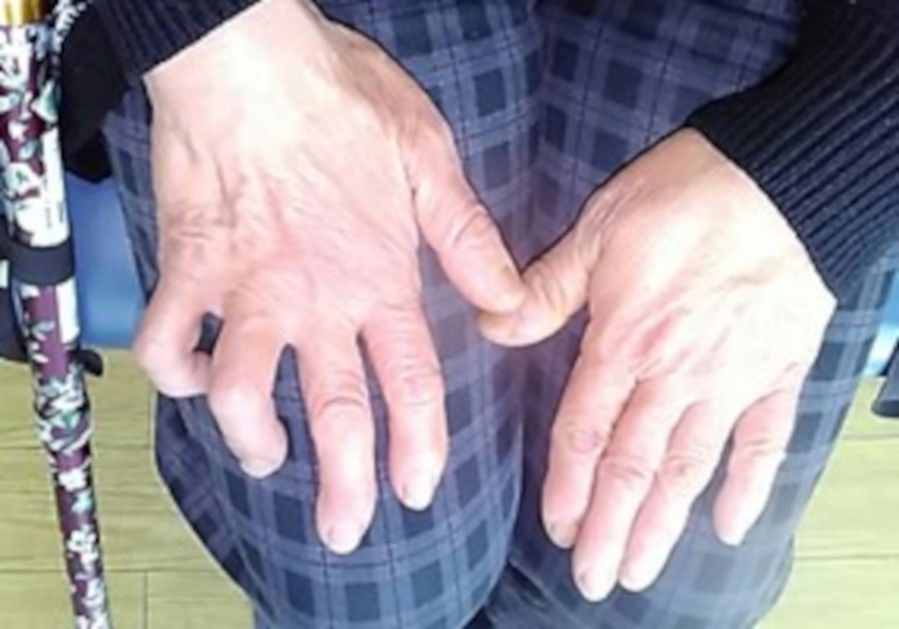
The patient had paralysis of the right fourth and fifth fingers, known as “claw hand”

**Fig. 2:**
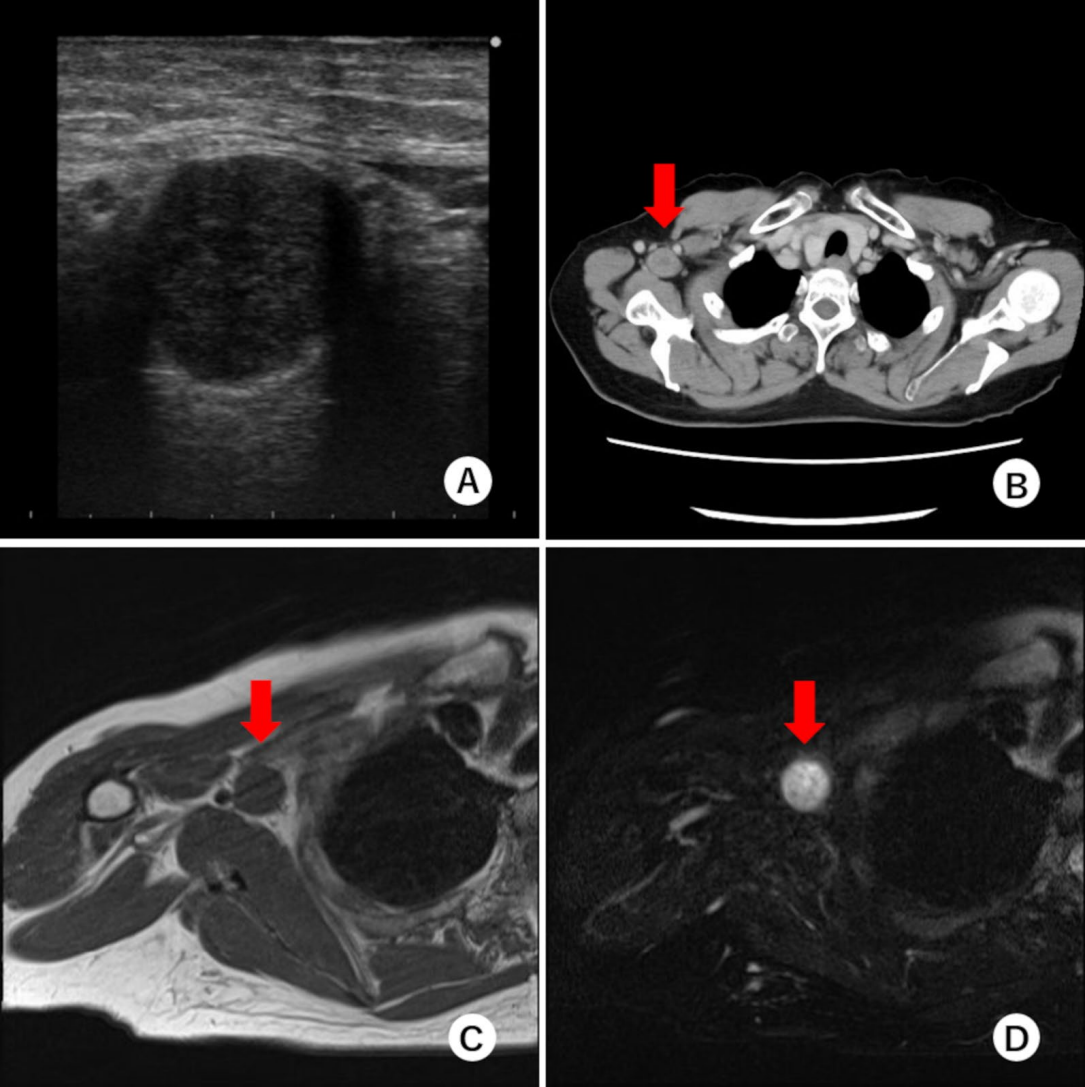
Imaging studies. **A** Ultrasonography revealed a well-defined hypoechoic tumor in the right axilla region; **B** enhanced computed tomography revealed a well-defined round tumor in the right axilla region (red arrow). The tumor was in contact with the axillary vein; **C**, **D** magnetic resonance imaging revealed a round tumor which had low intensity on T1-weighted images and high intensity on fat-saturation T2-weighted images in the right axilla region (red arrow)

**Fig. 3:**
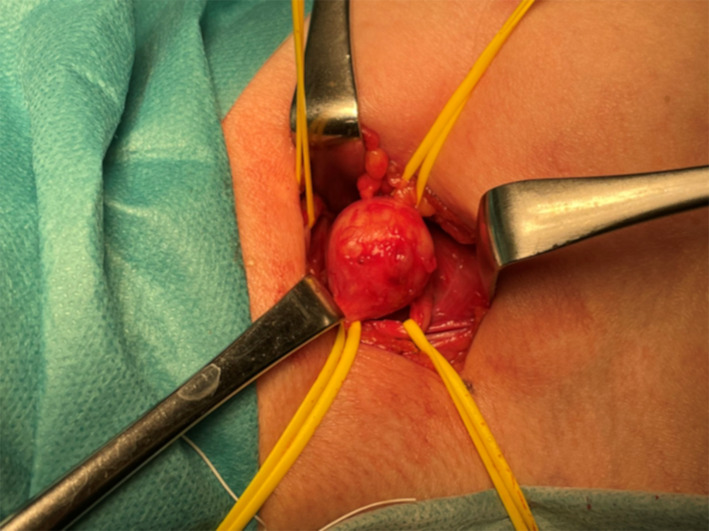
A tumor arising from the medial cord of the brachial plexus was observed intraoperatively

**Fig. 4:**
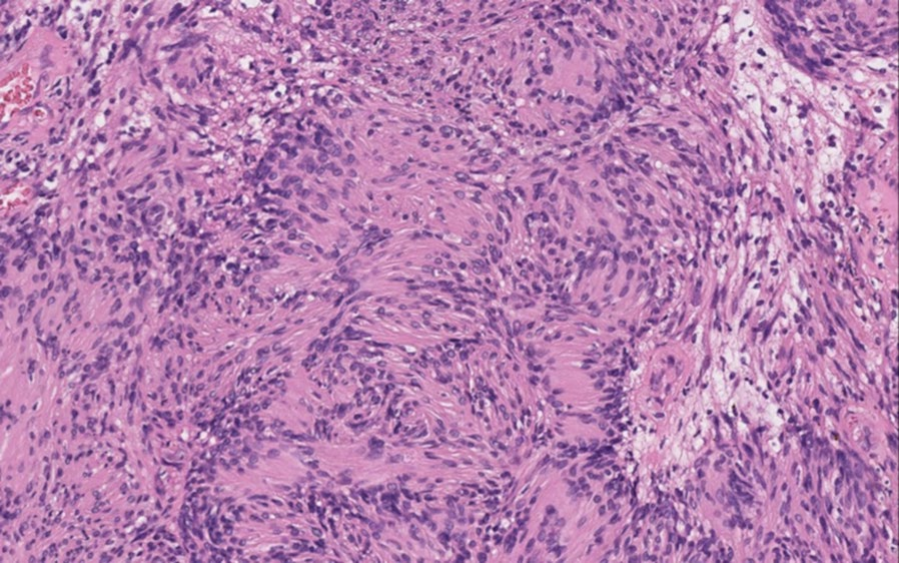
Histopathological finding (hematoxylin and eosin, ×200). Histopathological examination revealed typical findings of schwannoma, such as spindle-shaped cells with palisading nuclei and biphasic growth patterns (Antoni type A and B)

The patient underwent a mastectomy and axillary lymph node dissection for right breast cancer in May 1998. Histopathological findings revealed invasive ductal carcinoma, pT3b N2 (8/10) M0 stage IIIA. The tumor was positive for hormone receptors. The patient received adjuvant chemotherapy and hormone therapy postoperatively. In February 2021, when she was 68 years old, she presented to our hospital with numbness and weakness in the right arm for 6 months. She had no family history of neurofibromatosis. She had dysesthesia of the medial side of the right upper extremity and paralysis of the fourth and fifth fingers of the same side, known as “claw hand” (Fig. 1). The tumors were not palpable in the right axillary region, but the patient experienced radiating pain during the deep palpation (Tinel’s sign). Laboratory findings were unremarkable, and serum tumor marker levels, including carcinoembryonic antigen (CEA) and carbohydrate antigen 15-3 (CA 15-3), were within the normal range. Ultrasonography (US) demonstrated a 20-mm well-circumscribed hypoechoic tumor in the right axillary region (Fig. 2A). Computed tomography (CT) revealed a round tumor, which was well-circumscribed in the right axilla and next to the right axillary vein (Fig. 2B). Magnetic resonance imaging (MRI) also revealed a well-defined tumor with low intensity on T1-weighted images and high intensity on fat-saturation T2-weighted images (Fig. 2C, D). She was suspected to have axillary lymph node metastasis because of her history of advanced breast cancer. Initially, fine-needle aspiration (FNA) was performed, but the cytology was not diagnostic. Then, core needle biopsy (CNB) was performed to achieve an accurate diagnosis. The result of CNB did not confirm axillary lymph node metastasis, but schwannoma. When FNA and CNB were performed, the patient experienced a sharp radiating pain, which improved in a few days. She underwent tumor enucleation under a general anesthesia. Intraoperatively, the tumor was found to be derived from the medial cord of the brachial plexus (Fig. 3). Histopathological examination revealed a fibrous capsule and bundles of spindle-shaped cells with nuclear palisade. A compact hypercellular area (Antoni type A) and edematous hypocellular area (Antoni type B) were also observed. These findings were consistent with those of schwannoma (Fig. 4). Her weakness in the right upper limb worsened transiently after the operation, but it improved in the following 3 months. The patient had no recurrence 1 year postoperatively.

## Discussion

We report a unique case of axillary schwannoma mimicking lymph node metastasis. Furthermore, the present case highlighted the difficulty in making an accurate diagnosis of tumors in the axillary region and the importance of histopathological examination.

A great concern when examining patients with tumors in the axillary region with breast or other cancers is whether the tumor is lymph node metastasis or not. In particular, there are several problems when distinguishing schwannomas from lymph node metastasis. First, the two tumors resemble each other in imaging findings [4–8]. Schwannoma in the axillary region is very similar to swollen lymph nodes on CT [4] and has a low T1 signal and high T2 signal on MRI, as does lymph node metastasis [8]. In the present case, lymph node metastasis was initially suspected based on imaging findings. In addition, fluorodeoxyglucose (FDG)-positron emission tomography is a powerful modality for detecting malignant tumors, but FDG accumulation has also been observed in schwannomas [5, 9]. Therefore, distinguishing between schwannoma and lymph node metastasis by imaging studies alone is impractical. Second, cytology by FNA or CNB for tumors in the axillary region is often technically difficult because of anatomical problems; tumors in the axillary region are generally in contact with the axillary vasculature or nerves. There is a risk of iatrogenic vascular or nerve injury at the time of puncture. Therefore, some patients require an excisional biopsy to obtain an accurate diagnosis [5, 7, 8].

Tinel’s sign is a finding that is explained as a radiating pain caused by tapping over the affected nerve. This finding is characteristic of axillary schwannoma, and described in previous reports [7, 10]. In the present case, the patient also felt radiating pain on palpation. Furthermore, she felt a sharp and radiating pain when FNA and CNB were being performed. This sharp pain, felt during the tumor biopsy by FNA or CNB, is an invaluable finding suggesting that the tumor originates from the nervous system [7]. This finding can play a key role in diagnosing axillary schwannoma.

Making an accurate diagnosis of tumors in the axillary region is often difficult as mentioned above. However, clinicians should always make an effort to distinguish between lymph node metastasis and other tumors by histopathological examination, because the treatment strategies are completely different. Axillary schwannoma is a very rare tumor, but its clinical presentation and imaging findings generally mimic lymph node metastasis. When examining patients with a history of malignancy, especially breast cancer, and tumors in the axillary region, clinicians should consider the possibility of schwannoma and avoid chemotherapy or radiation therapy on the assumption that they have lymph node metastasis, without histopathological examinations.

In conclusion, axillary schwannomas often mimic lymph node metastasis in patients with a history of malignancy, particularly breast cancer. To select the optimal treatment, the clinicians should make as accurately as possible a diagnosis, with histopathological examinations, when examining patients with cancer who develop tumors in the axillary region.

## Data Availability

Not applicable.

## Abbreviations

CEA: Carcinoembryonic antigen
CA15-3: Carbohydrate antigen 15-3
US: Ultrasonography
CT: Computed tomography
MRI: Magnetic resonance imaging
FNA: Fine-needle aspiration
CNB: Core needle biopsy
FDG: Fluorodeoxyglucose
